# Global availability of medications and health technologies for kidney care: A multinational study from the ISN-GKHA

**DOI:** 10.1371/journal.pgph.0004268

**Published:** 2025-02-10

**Authors:** Dearbhla M. Kelly, Erika S. W. Jones, Zibya Barday, Silvia Arruebo, Fergus J. Caskey, Sandrine Damster, Jo-Ann Donner, Vivekanand Jha, Adeera Levin, Masaomi Nangaku, Syed Saad, Marcello Tonelli, Feng Ye, Ikechi G. Okpechi, Aminu K. Bello, David W. Johnson

**Affiliations:** 1 Adult Intensive Care Unit, John Radcliffe Hospital, Oxford University Hospitals NHS Foundation Trust, Oxford, United Kingdom; 2 Wolfson Centre for the Prevention of Stroke and Dementia, Nuffield Department of Clinical Neurosciences, University of Oxford, Oxford, United Kingdom; 3 Division of Nephrology and Hypertension, Department of Medicine, Groote Schuur Hospital, University of Cape Town, Western Cape, South Africa; 4 The International Society of Nephrology, Brussels, Belgium; 5 Population Health Sciences, Bristol Medical School, University of Bristol, Bristol, United Kingdom; 6 George Institute for Global Health, University of New South Wales (UNSW), New Delhi, India; 7 School of Public Health, Imperial College, London, United Kingdom; 8 Manipal Academy of Higher Education, Manipal, India; 9 Division of Nephrology, Department of Medicine, University of British Columbia, Vancouver, British Columbia, Canada; 10 Division of Nephrology and Endocrinology, The University of Tokyo Graduate School of Medicine, Tokyo, Japan; 11 Division of Nephrology and Immunology, Faculty of Medicine and Dentistry, University of Alberta, Edmonton, Alberta, Canada; 12 Department of Medicine, University of Calgary, Calgary, Alberta, Canada; 13 Canada and Pan-American Health Organization/World Health Organization’s Collaborating Centre in Prevention and Control of Chronic Kidney Disease, University of Calgary, Calgary, Alberta, Canada; 14 Division of Nephrology and Hypertension, University of Cape Town, Cape Town, South Africa; 15 Kidney and Hypertension Research Unit, University of Cape Town, Cape Town, South Africa; 16 Department of Kidney and Transplant Services, Princess Alexandra Hospital, Brisbane, Queensland, Australia; 17 Centre for Kidney Disease Research, University of Queensland at Princess Alexandra Hospital, Brisbane, Queensland, Australia; 18 Translational Research Institute, Brisbane, Queensland, Australia; 19 Australasian Kidney Trials Network at the University of Queensland, Brisbane, Queensland, Australia; London School of Economics and Political Science, UNITED KINGDOM OF GREAT BRITAIN AND NORTHERN IRELAND

## Abstract

A core feature of universal health coverage is equitable access to affordable care not exposing people to financial hardship. This study aims to provide a global overview on availability and access to medications and health technologies for delivery of optimal kidney care. An international survey of stakeholders (clinicians, policymakers, and patient advocates) from countries affiliated to the International Society of Nephrology was conducted from July to September 2022 on availability of tools and services for all aspects of kidney care and access to essential medications. Of 167 participating countries (97.4% of the global population), there were significant disparities in kidney care funding and service availability. Only 5 (n = 1) and 10% (n = 4) of countries in Latin America and Africa, respectively, publicly funded non-dialysis CKD care free at the point of delivery, compared to73% (n = 16) in Western Europe. Public funding (and free at point of delivery) for medications for dialysis and kidney transplantation was available in only 24% (n = 39) and 30% (n = 50) of countries worldwide, with the proportion increasing in line with country income levels. There was reduced capacity for the management of CKD mineral bone disease in low-income countries (LICs) - serum parathyroid hormone was available in only 26% (n = 5) of LICs and the ability to administer non-calcium-based phosphate binders and cinacalcet was also very limited in LICs [16% (n = 3) and 5% (n = 1), respectively]. Nutritional services like oral supplements were accessible in 32% (n = 6) of LICs versus 97% (n = 61) of high-income countries. This study highlights significant gaps in the global methods of funding and availability of medications, capacity for kidney disease monitoring, and capacity to treat complications of kidney disease to improve outcomes. Achieving universal and equitable access to essential medications and health technologies for kidney care is vital to tackle the rapidly growing global burden of kidney disease.

## Introduction

The International Society of Nephrology (ISN) advocates for universal health care for all people with kidney disease [1,2]. The World Health Organization (WHO) universal health coverage recommendations include the need to ensure equitable access to medications and health technologies in a way that do not put people seeking or receiving therapies at financial risk [2]. In the 2017 ISN Global Kidney Health Atlas (ISN-GKHA), a need for access to kidney function monitoring, dialysis, and transplantation was reported [3].

Worldwide, kidney disease is a major public health challenge with the rising prevalence of chronic kidney disease (CKD). The global prevalence of CKD rose from 10.4% in 2010 to 13.4% over a span of six years [4,5]. There are considerable differences in access to care depending on World Bank country classification (low- [LIC], lower middle- [LMIC], upper middle- [UMIC], and high-income countries [HIC]) [6]. Access to medications and technologies, for the management of people with kidney disease (both acute and chronic), has a considerable economic impact on health systems as well as individuals and their families. Access to, and reimbursement for these therapies differ widely between and within regions and countries [7].

Care of people living with CKD needs to include management of deficits in excretory and hormonal functions of the kidney [8]. Excretory functions include waste and drug removal, electrolyte balance, and fluid balance. The hormonal functions impact healthy bone mineralization, blood pressure stabilization, and the production of healthy red blood cells [9,10]. Progression of kidney disease associated with these disorders lead to various complications associated with excessive mortality and morbidity including impaired physical and cognitive function, poor quality of life, pruritus, fractures, fluid overload, higher frequency of hospitalizations, and an increase in cardiovascular events [11–14]. Capacity to monitor these disorders and availability of medicines to prevent or treat them is critical for improving outcomes in people living with kidney disease. A previous iteration of the ISN-GKHA identified significant gaps in capacity to monitor and availability of medicines to treat these complications, especially in LICs. Using recent data from the ISN-GKHA [15], we assessed the current methods of funding and availability of medications, capacity for disease monitoring, and capacity to treat complications associated with chronic kidney disease and kidney failure across countries.

## Methods

### Ethics statement

The University of Alberta Research Ethics Committee approved this project (protocol number: PRO00063121). Consent was not required by survey respondents. Our study did not report experiments on humans and/or the use of human tissue samples.

The ISN-GKHA is an international collaborative research initiative. The objective of this report was to collect data on global access to medication and health technology of kidney disease and kidney replacement therapy (KRT) including hemodialysis, peritoneal dialysis, and kidney transplantation. The methods of this study were structured into two components, 1) literature review and 2) a multi-national cross-sectional survey. This study included 218 countries recognized by the World Bank, with a focus on ISN affiliated countries, spanning over 10 ISN global regions: Africa, Eastern and Central Europe, Latin America, Middle East, Newly Independent States (NIS) and Russia, North America and the Caribbean, North and East Asia, Oceania and South East Asia (OSEA), South Asia, and Western Europe.

### Literature review

As per the previous ISN-GKHA iterations, a comprehensive literature review was designed by an expert research librarian [16]. Briefly, this included three sections, 1) a broad international literature review on national health system characteristics, 2) a review on the epidemiology of kidney disease, and 3) a scoping review on the worldwide KRT cost estimates. The broad literature review assessed the national health system characteristics based on each WHO universal health coverage domain. The domains included service delivery, health workforce, information systems, medicines and medical products, finance, and leadership [17]. Additional literature was identified from key stakeholders, such as national nephrology society leaders and ISN leaders, on the organization and delivery of kidney disease in their countries. This review served as a foundational step to gather existing evidence on national health systems, the epidemiology of kidney disease, and cost estimates for KRT. It identified broad trends and key gaps in existing knowledge, helping to frame the subsequent survey questions.

### Survey design

A multi-national cross-sectional survey was conducted by the ISN to assess the global capacity for KRT across various countries. The methods for this survey have been described in detail elsewhere [15,18]. Briefly, the survey was peer reviewed for content validity and comprehensiveness. The survey was piloted with the 10 ISN regional boards to identify any logistical and feasibility issues e.g., translational needs. The primary objective was to assess the global capacity, readiness, and governance of kidney care across countries. The survey assessed key areas aligned with the WHO universal health coverage domains including health finance and service delivery, health workforce for nephrology care, essential medications and health product access for kidney disease, health information systems and statistics, and leadership and governance. The content was developed with input from global kidney health experts, ISN leadership, and nephrology stakeholders to ensure comprehensiveness and validity. The survey was accompanied by a detailed information sheet about ISN-GKHA, instructions for completion and a glossary defining key terms used in the survey [15]. The survey was administered in English, French, and Spanish. Regional ISN leaders followed up survey responses for countries, reviewed regional data, and served as opinion leaders on regional projects.

The survey was conducted between June 1 to September 30, 2022. A non-probability, purposive sampling approach was employed to identify survey respondents. ISN national and regional leaders identified key stakeholders to conduct the survey, which included national nephrology society representatives, policymakers, patient organizations, foundations, and other advocacy groups. Respondents were specifically asked about heterogeneity within countries and to identify other respondents, to improve the quality of data captured.

In the survey, availability and methods of funding for medicines and various health technologies were assessed. “Generally available” was defined as being present in ≥50% while “Generally not available” was defined as being present in <50%. On completion of the surveys, data were collated on Microsoft Excel and merged to create a single global database. This was stored in a secure, centralized computer system with automated backups.

ISN regional leaders were responsible for ensuring that the collected data were consistent with their understandings and were of high quality. Each regional board leader reviewed information provided for countries within their region to clarify any ambiguity or inconsistencies and ensure that data provided were consistent with known information of the country. Any major inconsistencies that remained after the reviews were systematically addressed during follow-up inquiries with stakeholders involved with the survey. Further validation was carried out at the national and regional levels by triangulating the findings with published literature and gray sources of information (i.e., government reports and other sources provided by the survey respondents).

### Data handling and statistical analysis

The analysis of the data was conducted using STATA17 software [Stata Corporation using 2017]. The specified country was used as a unit of analysis, responses were summarized based on key survey domains using a descriptive statistical approach and reported as counts with percentages, or medians with interquartile ranges [IQR]. Results were stratified by ISN region and World Bank income group. The results were examined with emphasis on identification of key gaps and challenges across the various domains and reported in accordance with the Guidelines for Accurate and Transparent Health Estimates Reporting (GATHER) statement.

## Results

### Characteristics of participating countries

Of 191 countries approached, 167 participated (LICs: n = 20; 12.0%, LMICs: n = 45; 26.9%, UMICs: n = 39; 23.4%, and HICs: n = 63; 37.7%), comprising 97.4% of the world’s population. The percentage of gross domestic product spent on healthcare for each of these countries, classified by World Bank income groups, is presented in S1 Fig. Availability of services for kidney failure care varied worldwide.

### Funding systems for services and medications for non-dialysis CKD

**Fig 1:**
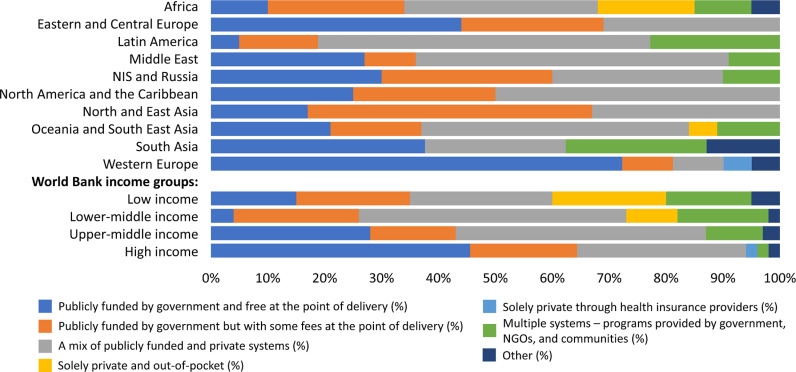
Funding models for non-dialysis chronic kidney disease, by ISN regions and World Bank income groups*. *Values represent absolute number of countries in each category expressed as a percentage of total number of countries. ISN, International Society of Nephrology; NIS, Newly Independent States; NGOs, non-governmental organizations.

There were significant regional variations in the funding mechanisms for non-dialysis CKD (Fig 1), including public funding with free services at the point of delivery, a mixture of public and private funding, and solely private funding and out-of-pocket payments. Government-funded and free at the point of delivery coverage for non-dialysis CKD care is highest in three ISN regions: Western Europe (n = 16; 73%), Eastern and Central Europe (n = 7; 44%), and South Asia (n = 3; 37%) (Fig 1). Approximately half of the countries in Latin America (n = 13; 59%), the Middle East (n = 6; 55%), and North America and the Caribbean (n = 6; 50%) regions financed non-dialysis CKD care through a mixture of public and private sources, with care either being provided free or requiring a co-payment at the point of delivery. A smaller proportion of countries in the ISN regions of Africa (n = 7; 17%) and OSEA (n = 1; 5%) relied exclusively on private funding and out-of-pocket payments for non-dialysis CKD. The exclusive use of private and out-of-pocket payment methods was reported in LICs (n = 4; 20%) and LMICs (n = 4; 9%), but not in other income groups. However, medications for people with CKD was publicly funded and free at point of delivery in 26 (16%) countries with the highest proportion being in Eastern and Central Europe (n = 7; 44%) while no countries in NIS and Russia and North and East Asia used this method to fund medications for people with CKD (S1 Table). There was a higher proportion of countries in South Asia (n = 3; 38%) and Africa (n = 13; 33%) where people with CKD not on dialysis paid for their medications solely privately and out-of-pocket. The proportion of countries where this method of payment was used decreased by income groups: LICs (n = 10; 53%), LMICs (n = 10; 22%), and UMICs (n = 2; 5%). There were no HICs where people with CKD not yet on dialysis paid for their medications solely out-of-pocket (S1 Table).

### Funding systems for services and medications for KRT (dialysis and kidney transplantation)

In countries where dialysis was available, medications were publicly funded by the government and free at the point of delivery in 24% (n = 39) of countries, publicly funded by the government with some fees at point of delivery in 28% (n = 47) of countries and privately funded and paid for completely out-of-pocket in 12% (n = 19) of countries (S2 Table). Notably, Eastern and Central Europe stood out as the ISN region where medications for people receiving dialysis were predominantly publicly funded by the government and provided free at the point of delivery, with 63% (n = 10) of countries adopting this model. Conversely, in Africa (n = 3; 8%) and OSEA (n = 1; 6%), the utilization of this payment approach is minimal (S2 Table). Countries relying exclusively on private and out-of-pocket payment systems were concentrated in Africa (n = 10; 25%), South Asia (n = 2; 25%), and NIS and Russia (n = 2; 20%).

**Fig 2:**
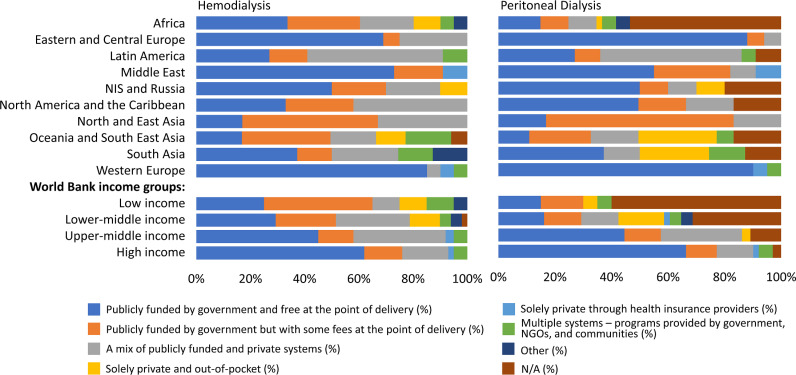
Funding models for care of people receiving dialysis (hemodialysis and peritoneal dialysis), by ISN regions and World Bank income groups*. *Values represent absolute number of countries in each category expressed as a percentage of total number of countries. ISN, International Society of Nephrology; NIS, Newly Independent States; NGOs, non-governmental organizations.

Hemodialysis was publicly funded and free at point of delivery in 74 (45%) of countries with more than half of countries in Western Europe (n = 19; 86%), the Middle East (n = 8; 73%), and Eastern and Central Europe using this method to fund hemodialysis services (Fig 2). The proportion of countries using this method increased by increasing country income levels: LICs (n = 5; 25%), LMICs (n = 13; 29%), UMICs (n = 17; 45%), and HICs (n = 39; 62%).

Peritoneal dialysis was publicly funded and free at point of delivery in 69 (42%) of countries worldwide. Similar to funding for hemodialysis, Western Europe (n = 20; 90%) and Eastern and Central Europe (n = 14; 88%) were the ISN regions where the highest proportion of countries used this method to fund peritoneal dialysis services (Fig 2).

Kidney transplant medications were publicly funded and free at the point of delivery in about a third of countries worldwide (n = 50; 30%). Other payment models for kidney transplantation medications included publicly funded by the government but with some fees at the point of delivery (n = 36; 22%), a mixture of public and private funding (n = 38; 23%), solely private and out-of-pocket (n = 23; 14%), solely private through health insurance (n = 4; 2%), and multiple systems of payment (n = 3; 2%). Only countries in three ISN regions used solely private and out of-pocket payment methods for kidney transplantation medications: Africa (n = 16; 40%), OSEA (n = 5; 28%), and South Asia (n = 2; 25%) (S3 Table). In more than half of the countries in Eastern and Central Europe (n = 9; 56%), the Middle East (n = 8; 73%), and the NIS and Russia (n = 7; 70%), the costs of medications for kidney transplantation were covered by the government and completely free at the point of delivery (S3 Table).

### Availability of services for monitoring CKD-related complications

Availability of an organized system and/or structures is necessary to ensure that people with established CKD are receiving guideline-concordant clinical care [3]. The study assessed the availability and capacity of such systems and/or structures to address various complications associated with CKD including anemia, CKD mineral bone disease, electrolyte disorders and metabolic acidosis, hypertension, and common kidney failure-associated symptoms such as uremic pruritus. The findings are presented in the following subsections.

### Management of hemoglobin level

**Fig 3:**
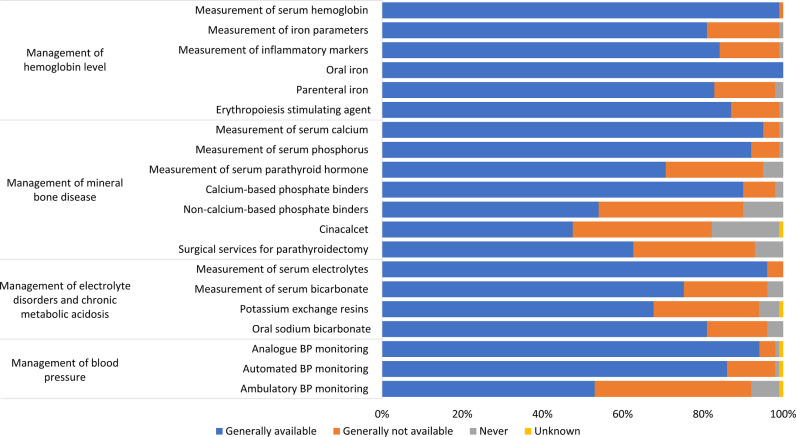
Availability of services for kidney failure care. BP, blood pressure.

All countries had the capacity to measure serum hemoglobin, and it was generally available in 99% (n = 163) of countries. Other services related to anemia management that were generally available included measurement of iron parameters [81% (n = 133) of countries], measurement of inflammatory markers [85% (n = 140) of countries], administration of oral iron [100% (n = 165) of countries], administration of parenteral iron (83% of countries), and use of erythropoiesis stimulating agents [87% (n = 144) of countries] (Fig 3). The capacity for measuring hemoglobin was available in 96% of LMICs and 100% (n = 120) of countries in other income groups. Moreover, as income levels increased, there was an associated rise in the ability to evaluate additional parameters relevant to anemia management (S2 Fig).

### Management of mineral bone disease

The ability to manage CKD mineral bone disease exhibited regional disparities. The majority of surveyed countries possessed the capacity to measure serum calcium (n = 157; 95%) and serum phosphate (n = 152; 92%), as well as to provide calcium-based phosphate binders (n = 149; 90%). A smaller proportion of countries had capacity to measure serum parathyroid hormone (n = 116; 70%), provide surgical services for parathyroidectomy (n = 103; 62%), or administer non-calcium-based phosphate binders (n = 89; 54%) and cinacalcet (n = 79; 48%) (Fig 3). Across all assessed parameters in the management of CKD mineral bone disease, UMICs and HICs had more diagnostic and therapeutic capabilities than countries at other income levels (S3 Fig). The capacity to measure serum parathyroid hormone was low among LICs (n = 5; 26%) and LMICs (n = 9; 47%), and the ability to administer non-calcium-based phosphate binders and cinacalcet was also very limited in LICs [16% (n = 3) and 5% (n = 1), respectively].

### Management of electrolyte disorders and chronic metabolic acidosis

The ability to measure serum electrolytes was present in the majority of countries (n = 158; 96%), and monitoring of acid-base balance, specifically serum bicarbonate, was available in 76% (n = 126) of countries. Potassium exchange resins were generally available in two-thirds of countries (n = 111; 67%) whereas oral sodium bicarbonate was widely available in 81% (n = 133) of countries (Fig 3). While the capacity to measure serum electrolytes was generally robust across countries of all income levels, LICs and LMICs had limited capacity to measure serum bicarbonate [32% (n = 6) and 58% (n = 11), respectively] as well as to provide potassium exchange resins [47% (n = 9) and 51% (n = 23), respectively] and oral sodium bicarbonate [32% (n = 6) and 76% (n = 34), respectively] (S4 Fig).

### Management of blood pressure

Analog equipment for blood pressure (BP) monitoring was generally available in 94% of countries and automated BP monitoring was generally available in 86% of countries. The capability to monitor BP using ambulatory BP monitoring (ABPM) equipment was generally available in 53% of countries (Fig 3). While the capacity to monitor BP using analogue or automated BP equipment was substantial across various income groups, the widespread availability of ABPM equipment showed an increasing trend with income level: LICs (n = 4; 21%), LMICs (n = 13; 29%), UMICs (n = 18; 47%), and HICs (n = 52; 83%) (S5 Fig).

### Management of common kidney failure-associated symptoms

Availability of management capacities for the treatment of other common symptoms of kidney failure (e.g., uremic pruritus and chronic pain) was also assessed. The widespread availability of gabapentinoids showed an upward trend with increasing income levels: LICs (n = 8 42%), LMICs (n = 22; 49%), UMICs (n = 19; 50%), and HICs (n = 58; 92%) (S6 Fig). A comparable pattern was evident concerning the general availability of non-morphine opioids, with LICs (n = 1; 5%) reporting notably limited availability compared to HICs (n = 52; 83%).

### Nutritional services

**Fig 4:**
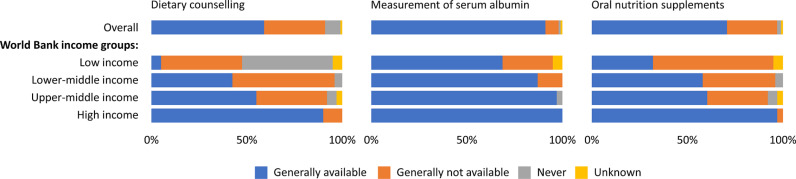
Availability of nutritional services for kidney care by World Bank income groups*. *Values represent absolute number of countries in each category expressed as a percentage of total number of countries.

The availability of nutritional services for kidney care was heterogenous worldwide. Dietary counselling was generally available in 59% (n = 98) of countries, while the availability of serum albumin measurement and oral nutrition supplements were noted in 92% (n = 152) and 70% (n = 116) of countries, respectively (Fig 4). The availability of nutritional services showed an upward trend corresponding to the income level of the country. Dietary counselling was available in only 5% (n = 1) of LICs compared to 42% (n = 19) of LMICs, 55% (n = 21) of UMICs, and 90% (n = 57) of HICs. Whereas measurement of serum albumin was generally available in all HICs, availability decreased with increasing country income level: HIC (n = 63; 100), UMICs (n = 37; 97%), LMICs (n = 39; 87%) and LICs (n = 13; 68%). Oral nutrition supplements were generally available in only 32% (n = 6) of LICs compared with 97% (n = 61) of HICs.

## Discussion

Universal access to healthcare services, including medications and technologies, is a fundamental goal outlined by the WHO and part of ISN mission [1,2]. However, this current iteration of the ISN-GKHA along with previous ones (2017, 2019) [7,16] reveal significant disparities in access to comprehensive kidney care across countries and income levels. The results of this study shed light on the global landscape of kidney care and highlight key findings that have implications for healthcare policy and resource allocation. Various guideline bodies, such as Kidney Disease Improving Global Outcomes, provide standards for the clinical evaluation and management of people with CKD or those receiving KRT, [3,19,20]. Our study description of the global, regional and country-level capacity for clinical evaluation and care enables us to assess readiness to meet these benchmarks.

The study underscores the importance of sustainable funding mechanisms in determining the accessibility and affordability of care for people with kidney diseases. Similar to the previous iteration of ISN-GKHA [3], financing for medications for non-dialysis CKD, dialysis, and recipients of kidney transplantation was government-funded and free at the point of delivery in less than half of the countries participating. Overall, non-dialysis CKD treatment is publicly funded by the government and free at the point of delivery in only 45 countries (27%). ISN regions of Western Europe, Eastern and Central Europe, and South Asia have the highest proportions of countries in which non-dialysis CKD treatment is publicly funded by the government and free at the point of delivery, which reflects health policy prioritization. Medications for people living with kidney failure are solely covered with private funding and paid for out-of-pocket in 13% of countries. Notably, only a minority of LICs and LMICs provide universal health coverage for all aspects of KRT.

Pre-dialysis care in particular is crucial for individuals with CKD to prevent or delay the progression of CKD to kidney failure, particularly in this era of new disease-modifying treatments including the ‘four pillars’ – sodium glucose co-transporter 2 inhibitors, glucagon-like peptide-1 receptor agonists, and the non-steroidal mineralocorticoid receptor antagonist finerenone, along with pre-existing angiotensin-converting enzyme inhibitors and angiotensin II receptor blockers [21]. This care encompasses both the use of such medications as well as regular monitoring of kidney function to minimize complications associated with the disease [22]. However, the costs associated with these essential components of CKD care can be prohibitive in countries where healthcare systems do not cover them, thereby contributing to a higher burden of kidney failure and health inequities, as the most vulnerable individuals may be unable to afford the necessary care [23]. An exemplar study from India showed that a significant proportion of people (40%) with non-dialysis dependent CKD experience catastrophic healthcare expenditure in the absence of universal healthcare that involved borrowing from family/friends, selling possessions, or taking out loans to fund healthcare [24]. Nonetheless, prioritizing access to pre-dialysis care and treatments could be a targeted policy intervention in low- and middle-income countries as a more cost-effective strategy than focusing on KRT [25,26].

Kidney failure is a complex disorder, and services to detect, monitor, and manage anemia, bone disease, electrolyte disorders, and metabolic acidosis are crucial for optimal care delivery, as recommended by Kidney Disease Improving Global Outcomes, the National Institute for Health and Care Excellence, and other guidelines [3,20,27]. Kidney Disease Improving Global Outcomes guidelines also provide recommendations on monitoring and managing these complications [3]. Recommendations are based on the stage of CKD, abnormalities detected and responses to therapies received. Monitoring and managing hypertension, mineral bone disease, anemia and electrolytes form the key aspects of caring for people with CKD. Despite this, medications for kidney care are largely not included on the WHO essential drugs list apart from oral iron [28]. Peritoneal dialysis solutions and erythropoiesis stimulating agents are only included on the complementary list.

While most countries have the capacity to monitor complications of kidney failure, not all countries have access to the full spectrum of options for treating these complications with higher-income countries typically having greater diagnostic and therapeutic capabilities. Key diagnostic tools included in the WHO essential diagnostics list include those required to manage people with CKD: hemoglobin, albumin, calcium, bicarbonate and other electrolytes, pH, creatinine, C reactive protein and iron studies. In this study, most countries were able to monitor hemoglobin levels with only two countries indicating that it was unavailable. 18% of all regions did not generally analyze iron status. Oral iron was available in all regions and parenteral iron was generally available in 83% of sites. 87% of countries had access to the use of erythropoiesis stimulating agents.

While capacity to measure serum calcium (95%) and serum phosphate (92%) is high worldwide, only 54% and 48% of countries are able to administer non-calcium-based phosphate binders and cinacalcet, respectively, to treat mineral bone disease. Similarly, LICs and LMICs are restricted in their capacity to measure serum bicarbonate, to prescribe potassium exchange resins and oral sodium bicarbonate, and to measure BP using ambulatory monitors. Access to gabapentinoids and non-morphine opioids to treat common kidney failure-associated symptoms, such as uremic pruritus and chronic pain, also varies significantly based on income level, with HICs reporting higher availability. Oral nutrition supplements are available in less than one third of LICs compared with nearly 100% of HICs. Such discrepancies highlight the need for health policies with targeted interventions to address gaps in care, particularly in low- and middle-income settings, to improve outcomes of people living with kidney disease.

In summary, there is clearly an impetus to optimize kidney care in specific regions or according to income levels in several ways. In low- and lower-middle-income countries, public funding for CKD care, especially pre-dialysis care should be prioritised as a cost-effective intervention. Existing universal health coverage frameworks should be leveraged to include CKD diagnostics and medications on essential health benefits packages. Collaboration with existing international organizations such as the WHO, ISN, and philanthropic foundations may help secure funding and technical assistance for improving CKD care infrastructure. It is necessary to support regional training and capacity building by investing in training programs for healthcare providers, especially in rural and underserved regions, to expand diagnostic and treatment capabilities. In middle-income countries, there may be urban-rural disparities that could be targeted by increasing healthcare resources in rural areas, such as telemedicine services for CKD monitoring. Domestic production of essential CKD medications and dialysis consumables could be incentivised to reduce costs and improve accessibility. National insurance schemes could be developed or expanded to cover CKD medications, diagnostics, and treatment. High-income countries should invest in CKD research, expand coverage for new therapies and promote multidisciplinary care models to increase access to integrated care teams for CKD management.

Strengths of this study include its comprehensive scope and large sample size. The study covered a wide range of aspects related to kidney disease care, including access to medications and technologies essential for the optimal management of CKD and its sequelae. This comprehensive approach allowed for a holistic assessment of the global landscape of kidney care. The study included data from 167 countries, making it one of the most extensive assessments of kidney care on a global scale. The large sample size enhanced the study’s representativeness and generalizability. Furthermore, by employing a combination of literature reviews and a multi-national cross-sectional survey, the study used a mixed-methods approach to collect data. This approach helped to triangulate information from various sources, increasing the reliability of the findings. In addition, to date, the advocacy around kidney care has mainly focused on screening/case finding with little attention to access to care after diagnosis. This is the first study to provide a scoping landscaping of these domains.

However, this study also has a number of limitations. First, the accuracy and completeness of data may have varied across countries, potentially leading to gaps or inaccuracies in the findings. Second, the survey portion of the study relied on responses from various stakeholders, including policymakers and healthcare professionals. There may have been response bias, as individuals may have provided information that reflected positively on their countries or organizations. Third, while this study highlighted disparities in access to kidney care based on income levels and ISN regions, it may not have fully accounted for the complex economic, political, and cultural factors that influence healthcare access. Fourth, the analyses presented here may not have reflected patient-centered outcomes and experiences, hence, the preferences of people with kidney disease are crucial for understanding the quality of care. Fifth, we did not have access to data regarding diagnostics and management of acute kidney injury and thus, we were unable to discuss this aspect of global kidney care.

Future iterations could overcome some of these limitations by expanding languages for surveys to ensure inclusivity and reduce barriers to participation in non-native language regions and by complementing stakeholder surveys with community-level data collection to capture granular insights, especially in regions with significant intra-country heterogeneity. We could gather inputs from a wider variety of stakeholders, including frontline healthcare workers and patients, to balance the perspectives of policymakers or national representatives. We may attempt to disaggregate data by subgroups (e.g. women vs men; rural vs urban healthcare settings) to better capture inequities. We will endeavour to collect data on patient-reported outcomes (e.g., quality of life, satisfaction with care) to complement system-level metrics. Future surveys may also expand the focus to AKI and include specific questions on AKI diagnostics, treatment practices, and outcomes in future surveys.

## Conclusion

In conclusion, the study provides a comprehensive overview of the state of kidney disease care globally, highlighting variations in access, funding mechanisms, and service availability. Achieving universal access to essential medications, technologies, and support services for CKD management is essential to mitigate the growing burden of kidney disease on individuals and healthcare systems. Collaborative initiatives, policy reforms, and increased investment in healthcare infrastructure are necessary steps to advance the goal of universal kidney care and align with the principles of universal health coverage advocated by the ISN and the WHO. Efforts should focus largely on improving public funding for all domains of kidney care, especially in low-resource settings. Addressing these disparities will not only improve the lives of people with CKD but also contribute to the overall health and well-being of populations globally.

## Supporting information

S1 Fig Healthcare expenditure as percentage GDP for participating countries classified by World Bank income groups. (PDF)

S2 Fig Management of hemoglobin level by World Bank income groups. (PDF)

S3 Fig Management of chronic kidney disease mineral bone disease by World Bank income groups. (PDF)

S4 Fig Management of electrolyte disorders and chronic metabolic acidosis by World Bank income groups. (PDF)

S5 Fig Management of blood pressure by World Bank income groups. (PDF)

S6 Fig Management of common kidney failure symptoms by World Bank income groups. (PDF)

S1 Table Funding of medications for people with CKD (not on dialysis), by ISN regions and World Bank income groups (N, %). (PDF)

S2 Table Funding of medications for all people on dialysis, by ISN regions and World Bank income groups (N, %). (PDF)

S3 Table Funding of medications for all recipients of kidney transplants, by ISN regions and World Bank income groups (N, %). (PDF)

S1 Checklist Inclusivity in global research. (PDF)

S1 Data Medications and health tech. (XLSX)
