# Supplementary material for: Global availability of medications and health technologies for kidney care: A multinational study from the ISN-GKHA
Source: PLOS Glob Public Health. 2025 Feb 10;5(2):e0004268. doi: 10.1371/journal.pgph.0004268 (PMC11809785; doi:10.1371/journal.pgph.0004268)
Supplement: S1 Fig — (PDF) [file pgph.0004268.s001.pdf]

S1 Fig. Healthcare expenditure as percentage GDP* for participating countries classified by World Bank income groups.

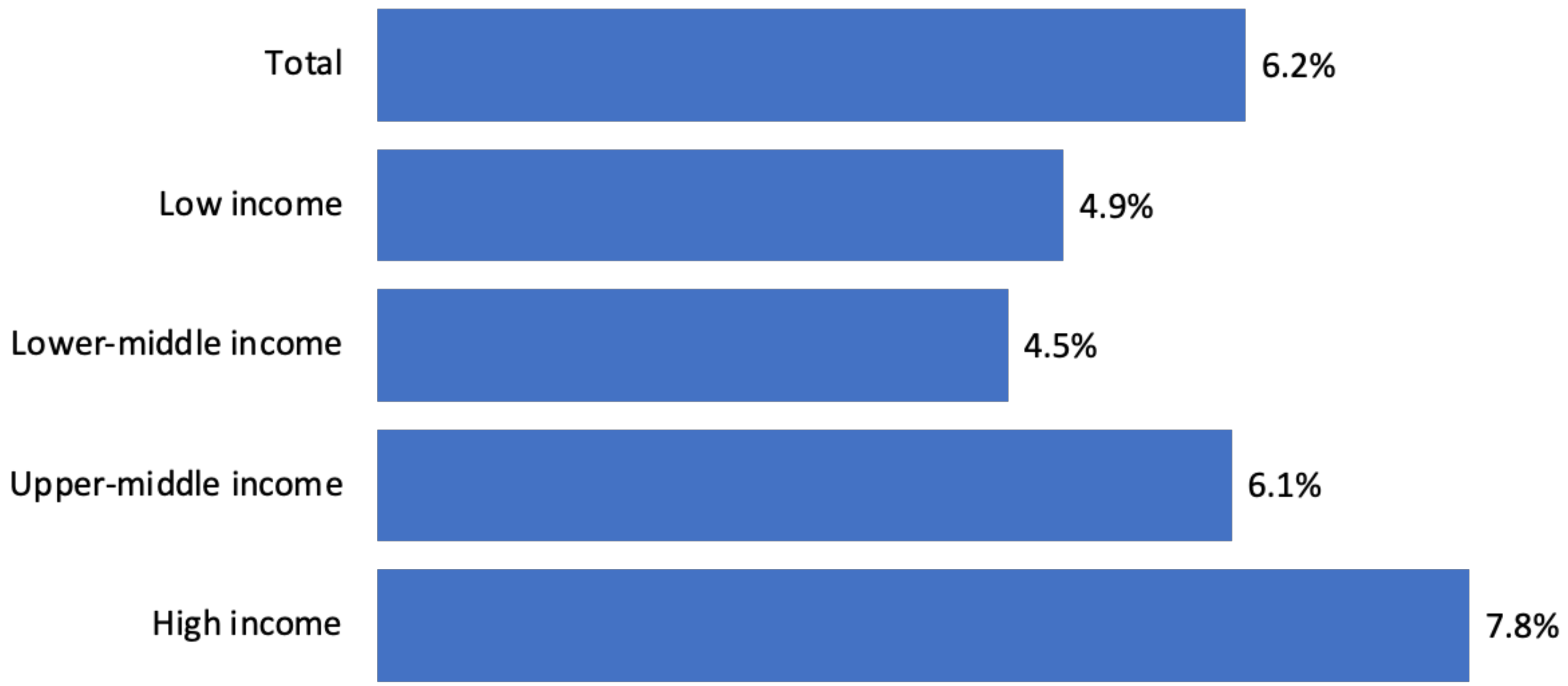

Abbreviation: GDP = gross domestic product
*Data from: The Institute for Health Metrics and Evaluation. Global Health Data Exchange. Global Expected Health Spending 2019-2050. [Accessed April 8, 2022]. Available from: https://ghdx.healthdata.org/record/ihme-data/global-expected-health-spending-2019-2050.
