# Supplementary material for: Global availability of medications and health technologies for kidney care: A multinational study from the ISN-GKHA
Source: PLOS Glob Public Health. 2025 Feb 10;5(2):e0004268. doi: 10.1371/journal.pgph.0004268 (PMC11809785; doi:10.1371/journal.pgph.0004268)
Supplement: S2 Fig — (PDF) [file pgph.0004268.s002.pdf]

S2 Fig. Management of hemoglobin level by World Bank income groups.*

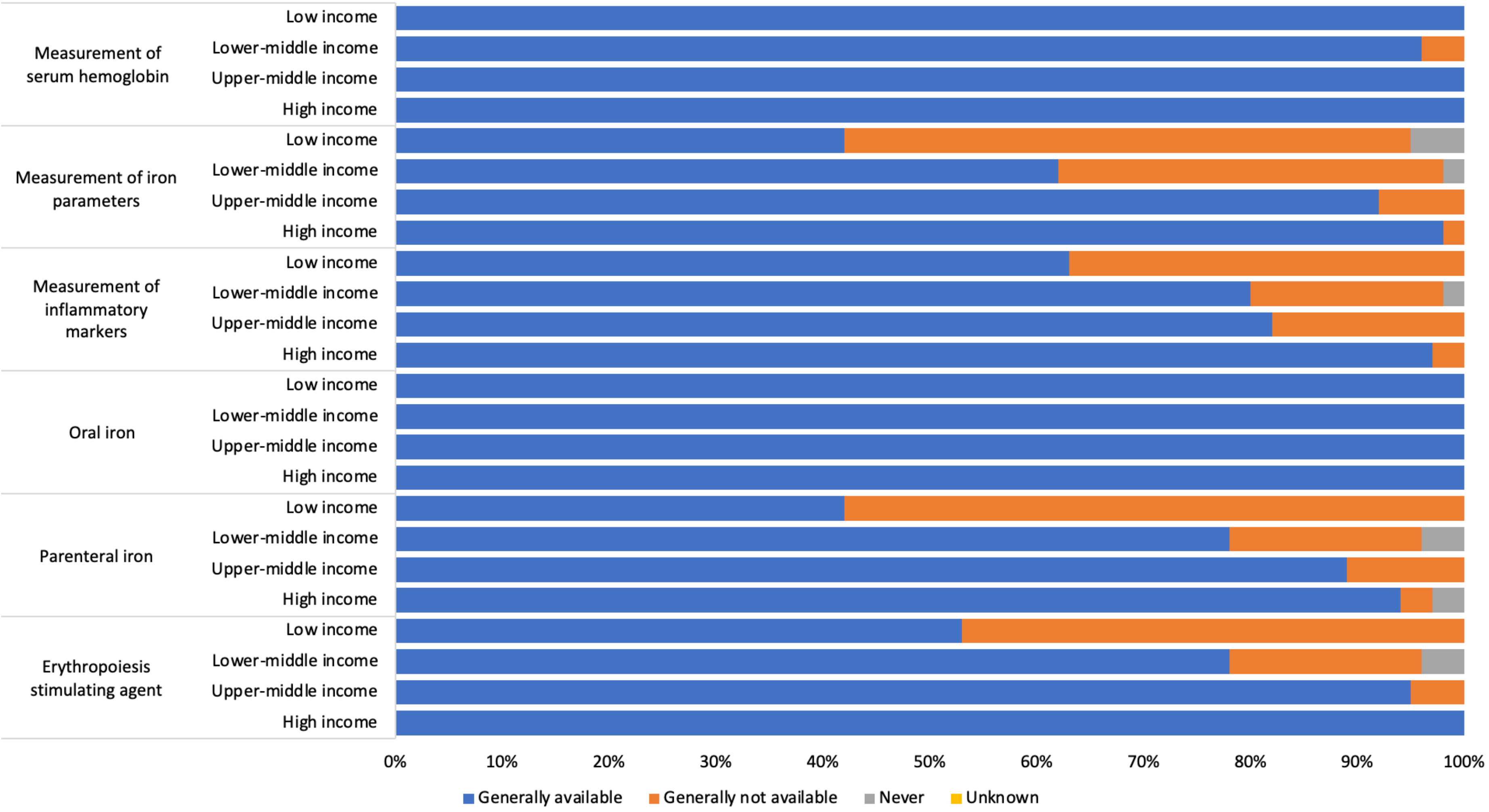

*Values represent absolute number of countries in each category expressed as a percentage of total number of countries.
