# Supplementary material for: Global availability of medications and health technologies for kidney care: A multinational study from the ISN-GKHA
Source: PLOS Glob Public Health. 2025 Feb 10;5(2):e0004268. doi: 10.1371/journal.pgph.0004268 (PMC11809785; doi:10.1371/journal.pgph.0004268)
Supplement: S1 Table — (PDF) [file pgph.0004268.s007.pdf]

S1 Table. Funding of medications for people with CKD (not on dialysis), by ISN regions and World Bank income groups (N, %).

	Publicly funded by government and free at the point of delivery	Publicly funded by government but with some fees at the point of delivery	A mix of publicly funded (whether or not publicly funded component is free at point of delivery) and private systems	Solely private and out-of-pocket	Solely private through health insurance providers	Multiple systems - programs provided by government, NGOs, and communities	Other (please specify)	Total
**Overall**	26 (16)	54 (33)	47 (28)	22 (13)	4 (2)	11 (7)	1 (1)	165
**ISN regions:**								
Africa	3 (8)	7 (18)	14 (35)	13 (33)	0 (0)	2 (5)	1 (3)	40
Eastern and Central Europe	7 (44)	9 (56)	0 (0)	0 (0)	0 (0)	0 (0)	0 (0)	16
Latin America	2 (9)	5 (23)	11 (50)	1 (5)	0 (0)	3 (14)	0 (0)	22
Middle East	4 (36)	3 (27)	2 (18)	1 (9)	0 (0)	1 (9)	0 (0)	11
NIS and Russia	0 (0)	5 (50)	0 (0)	2 (20)	1 (10)	2 (20)	0 (0)	10
North America and the Caribbean	1 (8)	3 (25)	7 (58)	1 (8)	0 (0)	0 (0)	0 (0)	12
North and East Asia	0 (0)	5 (83)	1 (17)	0 (0)	0 (0)	0 (0)	0 (0)	6
Oceania and South East Asia	3 (17)	5 (28)	8 (44)	1 (6)	0 (0)	1 (6)	0 (0)	18
South Asia	2 (25)	0 (0)	2 (25)	3 (38)	0 (0)	1 (13)	0 (0)	8
Western Europe	4 (18)	12 (55)	2 (9)	0 (0)	3 (14)	1 (5)	0 (0)	22
**World Bank income groups:**								
Low income	2 (11)	3 (16)	2 (11)	10 (53)	0 (0)	1 (5)	1 (5)	19
Lower-middle income	4 (9)	9 (20)	18 (40)	10 (22)	0 (0)	4 (9)	0 (0)	45
Upper-middle income	7 (18)	12 (32)	12 (32)	2 (5)	1 (3)	4 (11)	0 (0)	38
High income	13 (21)	30 (48)	15 (24)	0 (0)	3 (5)	2 (3)	0 (0)	63

Abbreviations: CKD = chronic kidney disease; ISN = International Society of Nephrology; NIS = Newly Independent States; NGOs = non-governmental organizations
