# Supplementary material for: Global availability of medications and health technologies for kidney care: A multinational study from the ISN-GKHA
Source: PLOS Glob Public Health. 2025 Feb 10;5(2):e0004268. doi: 10.1371/journal.pgph.0004268 (PMC11809785; doi:10.1371/journal.pgph.0004268)
Supplement: S2 Table — (PDF) [file pgph.0004268.s008.pdf]

S2 Table. Funding of medications for all people on dialysis, by ISN regions and World Bank income groups (N, %).

	Publicly funded by government and free at the point of delivery	Publicly funded by government but with some fees at the point of delivery	A mix of publicly funded (whether or not publicly funded component is free at point of delivery) and private systems	Solely private and out-of-pocket	Solely private through health insurance providers	Multiple systems - programs provided by government, NGOs, and communities	Other (please specify)	Total
**Overall**	39 (24)	47 (28)	47 (28)	19 (12)	2 (1)	9 (5)	2 (1)	165
**ISN regions:**								
Africa	3 (8)	8 (20)	16 (40)	10 (25)	0 (0)	2 (5)	1 (3)	40
Eastern and Central Europe	10 (63)	6 (38)	0 (0)	0 (0)	0 (0)	0 (0)	0 (0)	16
Latin America	4 (18)	5 (23)	11 (50)	1 (5)	0 (0)	1 (5)	0 (0)	22
Middle East	5 (45)	3 (27)	2 (18)	1 (9)	0 (0)	0 (0)	0 (0)	11
NIS and Russia	4 (40)	2 (20)	1 (10)	2 (20)	0 (0)	1 (10)	0 (0)	10
North America and the Caribbean	2 (17)	2 (17)	7 (58)	1 (8)	0 (0)	0 (0)	0 (0)	12
North and East Asia	1 (17)	4 (67)	1 (17)	0 (0)	0 (0)	0 (0)	0 (0)	6
Oceania and South East Asia	1 (6)	6 (33)	5 (28)	2 (11)	0 (0)	3 (17)	1 (6)	18
South Asia	2 (25)	1 (13)	2 (25)	2 (25)	0 (0)	1 (13)	0 (0)	8
Western Europe	7 (32)	10 (45)	2 (9)	0 (0)	2 (9)	1 (5)	0 (0)	22
**World Bank income groups:**								
Low income	2 (11)	2 (11)	4 (21)	9 (47)	0 (0)	1 (5)	1 (5)	19
Lower-middle income	4 (9)	11 (24)	17 (38)	8 (18)	0 (0)	4 (9)	1 (2)	45
Upper-middle income	14 (37)	8 (21)	13 (34)	2 (5)	0 (0)	1 (3)	0 (0)	38
High income	19 (30)	26 (41)	13 (21)	0 (0)	2 (3)	3 (5)	0 (0)	63

Abbreviations: ISN = International Society of Nephrology; NIS = Newly Independent States; NGOs = non-governmental organizations
