# Supplementary material for: Global availability of medications and health technologies for kidney care: A multinational study from the ISN-GKHA
Source: PLOS Glob Public Health. 2025 Feb 10;5(2):e0004268. doi: 10.1371/journal.pgph.0004268 (PMC11809785; doi:10.1371/journal.pgph.0004268)
Supplement: S3 Table — (PDF) [file pgph.0004268.s009.pdf]

S3 Table. Funding of medications for all recipients of kidney transplants, by ISN regions and World Bank income groups (N, %).

	Publicly funded by government and free at the point of delivery	Publicly funded by government but with some fees at the point of delivery	A mix of publicly funded (whether or not publicly funded component is free at point of delivery) and private systems	Solely private and out-of-pocket	Solely private through health insurance providers	Multiple systems - programs provided by government, NGOs, and communities	Other (please specify)	Total
**Overall**	50 (30)	36 (22)	38 (23)	23 (14)	4 (2)	3 (2)	11 (7)	165
**ISN regions:**								
Africa	4 (10)	5 (13)	9 (23)	16 (40)	1 (3)	0 (0)	5 (13)	40
Eastern and Central Europe	9 (56)	7 (44)	0 (0)	0 (0)	0 (0)	0 (0)	0 (0)	16
Latin America	8 (36)	3 (14)	9 (41)	0 (0)	0 (0)	1 (5)	1 (5)	22
Middle East	8 (73)	1 (9)	2 (18)	0 (0)	0 (0)	0 (0)	0 (0)	11
NIS and Russia	7 (70)	3 (30)	0 (0)	0 (0)	0 (0)	0 (0)	0 (0)	10
North America and the Caribbean	1 (8)	2 (17)	7 (58)	0 (0)	0 (0)	0 (0)	2 (17)	12
North and East Asia	1 (17)	4 (67)	1 (17)	0 (0)	0 (0)	0 (0)	0 (0)	6
Oceania and South East Asia	2 (11)	3 (17)	6 (33)	5 (28)	0 (0)	0 (0)	2 (11)	18
South Asia	2 (25)	0 (0)	2 (25)	2 (25)	0 (0)	1 (13)	1 (13)	8
Western Europe	8 (36)	8 (36)	2 (9)	0 (0)	3 (14)	1 (5)	0 (0)	22
**World Bank income groups:**								
Low income	3 (16)	3 (16)	0 (0)	9 (47)	1 (5)	0 (0)	3 (16)	19
Lower-middle income	7 (16)	4 (9)	15 (33)	13 (29)	0 (0)	1 (2)	5 (11)	45
Upper-middle income	18 (47)	7 (18)	10 (26)	1 (3)	0 (0)	0 (0)	2 (5)	38
High income	22 (35)	22 (35)	13 (21)	0 (0)	3 (5)	2 (3)	1 (2)	63

Abbreviations: ISN = International Society of Nephrology; NIS = Newly Independent States; NGOs = non-governmental organizations
